# Acidic/Alkaline Stress Mediates Responses to Azole Drugs and Oxidative Stress in Aspergillus fumigatus

**DOI:** 10.1128/spectrum.01999-21

**Published:** 2022-02-23

**Authors:** Jinxing Song, Landan Shi, Sha Wang, Yunqiu Wang, Yi Zhu, Jihong Jiang, Rongpeng Li

**Affiliations:** a The Key Laboratory of Biotechnology for Medicinal Plants of Jiangsu Province and School of Life Science, Jiangsu Normal University, Xuzhou, Jiangsu, China; b Key Laboratory of Vector Biology and Pathogen Control of Zhejiang Province, Huzhou Central Hospital, Huzhou University, Huzhou, Zhejiang, China; Broad Institute

**Keywords:** acidic/alkaline, *Aspergillus fumigatus*, azole drugs, oxidative stress

## Abstract

A human host exploits stresses such as acidic/alkaline pH, antifungal drugs, and reactive oxygen species to kill microbial pathogens such as the fungus Aspergillus fumigatus. However, A. fumigatus is resistant to these stresses *in vitro*. Therefore, what accounts for the potent antifungal activity of the human host? In this observation, we show that simultaneous exposure to acidic pH and oxidative stresses is much more potent than the individual stresses themselves and that this combinatorial stress kills A. fumigatus synergistically *in vitro*. Interestingly, A. fumigatus is resistant to the combination of alkaline pH and oxidative stress. Quantitative real-time PCR analyses showed that acidic/alkaline pH stress can mediate oxidative stress responses in A. fumigatus by regulating the expression of catalase-encoding genes. We further show that A. fumigatus is sensitive to the combination of acidic/alkaline stress and azole drug stress. Transcriptome analysis revealed that the sensitivity of A. fumigatus to azole drugs under acidic/alkaline conditions may be related to changes in genetic stability, sphingolipid metabolism, lipid metabolism, and amino acid metabolism. Collectively, our findings suggest that combinatorial stress represents a powerful fungicidal mechanism employed by hosts against pathogens, which suggests novel approaches to potentiate antifungal therapy.

**IMPORTANCE** The human host combats fungal infections via phagocytic cells that recognize and kill fungal pathogens. Immune cells combat Aspergillus fumigatus infections with a potent mixture of chemicals, including reactive oxygen species, acidic/alkaline stress, and antifungal drugs. However, A. fumigatus is relatively resistant to these stresses *in vitro*. In this observation, we show that it is the combination of acidic/alkaline pH and oxidative or azole stress that kills A. fumigatus so effectively, and we define the molecular mechanisms that underlie this potency. Our findings suggest that combinatorial stress is a powerful fungicidal mechanism employed by hosts, which suggests novel approaches to potentiate antifungal therapy. This study provides a platform for future studies that will address the combinatorial impacts of various environmental stresses on A. fumigatus and other pathogenic microbes.

## OBSERVATION

The filamentous fungus Aspergillus fumigatus is the most prevalent airborne fungal pathogen of humans. It causes severe invasive aspergillosis in immunocompromised patients (1). A. fumigatus strains are able to produce an enormous number of conidia (2), and humans are incidentally infected by inhalation of small numbers of spores. The first step in induction of the antifungal innate immune response depends on recognition of conserved fungal structures by front-line immune cells, including neutrophils and macrophages (3). Following recognition of A. fumigatus conidia, phagocytes initiate engulfment, leading to the formation of a phagosome (3). This vesicle then undergoes a series of fissions and fusions, ultimately leading to the formation of the phagolysosome. The degradative and microbicidal microenvironment of this organelle is associated with low pH, the generation of reactive oxygen species, and the presence of defensins and hydrolytic enzymes (3, 4). Moreover, antifungal drugs such as azoles are often used to exert stress on the cell membrane of A. fumigatus (5). Thus, human phagocytic cells combat A. fumigatus infections by killing this fungus with a potent mixture of these stresses. *In vitro*, A. fumigatus exhibits robust adaptation to low-pH stress, oxidative stress, and antifungal drug stress. Therefore, we reasoned that the potency of phagocytic cells might be due to a synergistic combination of these stresses.

One attribute of A. fumigatus that makes it such a successful opportunistic pathogen is its ability to adapt to, and proliferate in, a broad range of host environments. One of the most important environmental conditions that fluctuates between niches is the ambient pH (4, 6). A. fumigatus is able to grow in media from pH 3.5 to pH 10 (7).

In this report, we describe the effects of the combination of acidic/alkaline pH stress with oxidative stress or azole stress on the viability of A. fumigatus *in vitro*, revealing that this fungus is extremely sensitive to these combined stresses, and then we dissect the mechanistic basis for this extreme sensitivity. To date, the impact of these combinatorial stresses on cell viability has not been described.

**FIG 1:**
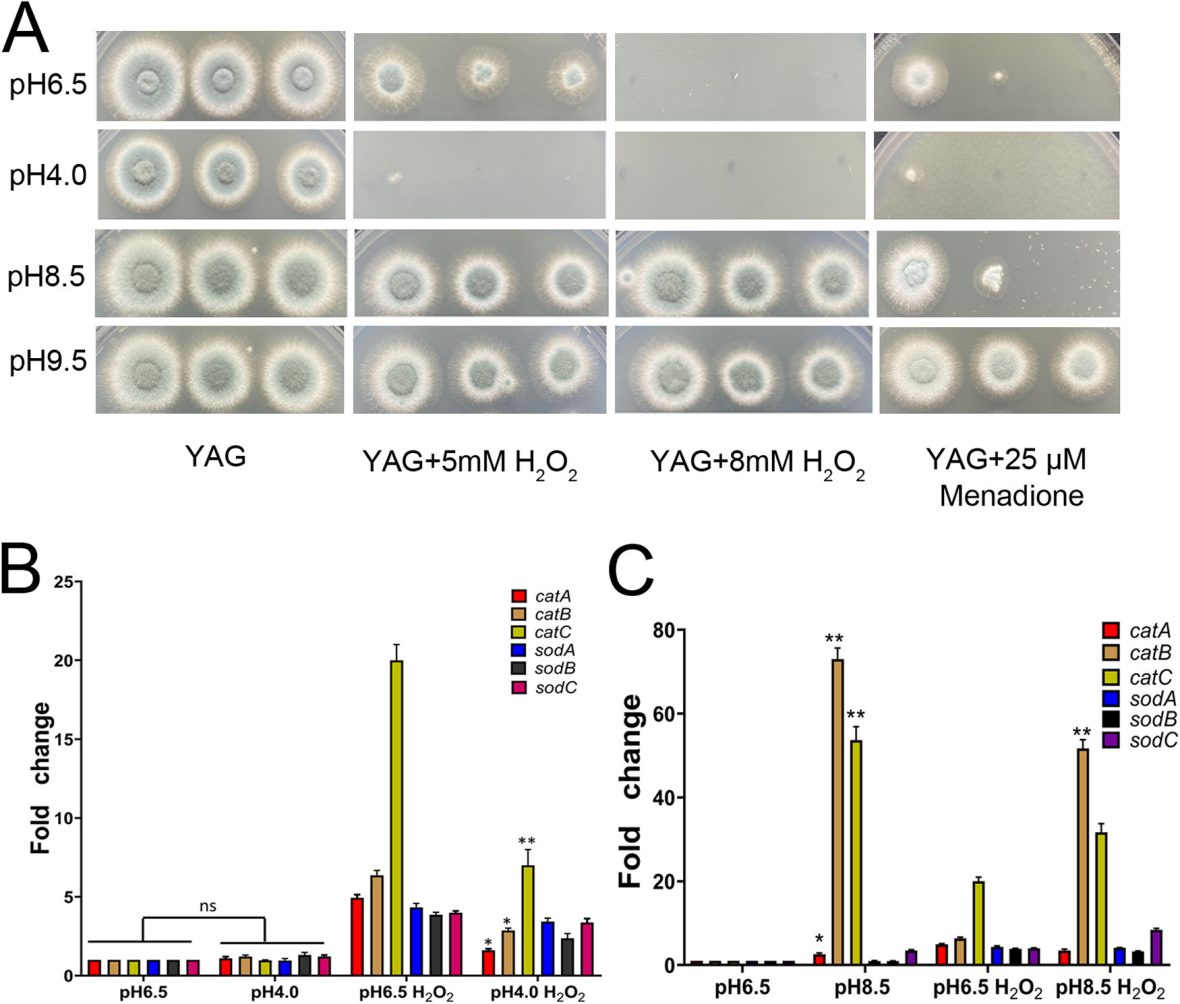
Acidic/alkaline pH stress affects the sensitivity of A. fumigatus to oxidative stress. (A) A. fumigatus strains were inoculated as a series of 3-μL 10-fold dilutions derived from a starting suspension of 10^7^ conidia per mL onto solid YAG medium (pH 6.5, pH 4.0, pH 8.5, or pH 9.5) with or without H_2_O_2_ or menadione and were cultured at 37°C for 2 days. (B) Analysis of the acidic pH stress impact on transcript levels of genes involved in ROS detoxification. Quantitative real-time reverse transcription (RT)-PCR of *catA/B/C* and *sodA/B/C* transcript levels in A. fumigatus strains under the combination of acidic pH stress and H_2_O_2_ stress was performed. (C) Analysis of the alkaline pH stress impact on transcript levels of genes involved in ROS detoxification. Quantitative real-time RT-PCR of *catA/B/C* and *sodA/B/C* transcript levels in A. fumigatus strains under the combination of alkaline pH stress and H_2_O_2_ stress was performed. Gene expression was normalized to the endogenous reference gene *tubA*. Experiments were carried out in triplicate. Values are reported as the means ± standard errors of the means (SEMs). Statistical significance was calculated using the unpaired two-tailed *t* test. ns, not significant; *, *P < *0.05; **, *P < *0.01.

First, we tested the levels of killing by a combination of acidic/alkaline pH and oxidative stresses in complete medium (yeast extract-agar-glucose [YAG] medium). As shown in Fig. 1A, A. fumigatus cells were killed synergistically by the combination of oxidative stress plus acidic stress. Interestingly, A. fumigatus was resistant to the combination of alkaline pH and oxidative stress. Furthermore, the addition of menadione to the medium at pH 8.5 or 9.5 had different inhibitory effects. When the alkalinity in the medium was stronger, the resistance of A. fumigatus to menadione was greater. Next, we carried out the experiment in minimal medium (MM), and our results showed that the phenotype in MM was consistent with that in YAG medium (see Fig. S1A in the supplemental material). Taken together, these results indicate that the oxidative stress resistance of A. fumigatus might be alkaline dependent. Moreover, like in YAG medium, A. fumigatus cells were killed synergistically by the combination of oxidative stress plus acidic stress in MM (see Fig. S1B), suggesting that A. fumigatus has the same phenotype on YAG medium and MM with specific pH values. Superoxide dismutase (SOD) and catalase (CAT) are enzymes that help cells resist oxidative damage (8–11); SOD converts superoxide anion free radicals into hydrogen peroxide (H_2_O_2_) and oxygen, while CAT detoxifies H_2_O_2_ into oxygen and water. To investigate the mechanistic basis of the effects of the combined stresses, we analyzed and compared the transcription levels of the genes *catA*, *catB*, *catC*, *sodA*, *sodB*, and *sodC* by real-time-PCR, using tubulin as the reference gene. As shown in Fig. 1B, under oxidative stress the transcription levels of *catA*, *catB*, and *catC* significantly decreased in A. fumigatus treated with acid (pH 4.0), compared with A. fumigatus at normal pH (pH 6.5). Interestingly, the transcription levels of *catA*, *catB*, and *catC* significantly increased in A. fumigatus treated with alkali (pH 8.5), compared with A. fumigatus at normal pH (Fig. 1C). Taken together, these results suggest that acidic/alkaline pH stress mediates oxidative stress responses in A. fumigatus by regulating the expression of *catA/B/C*.

**FIG 2:**
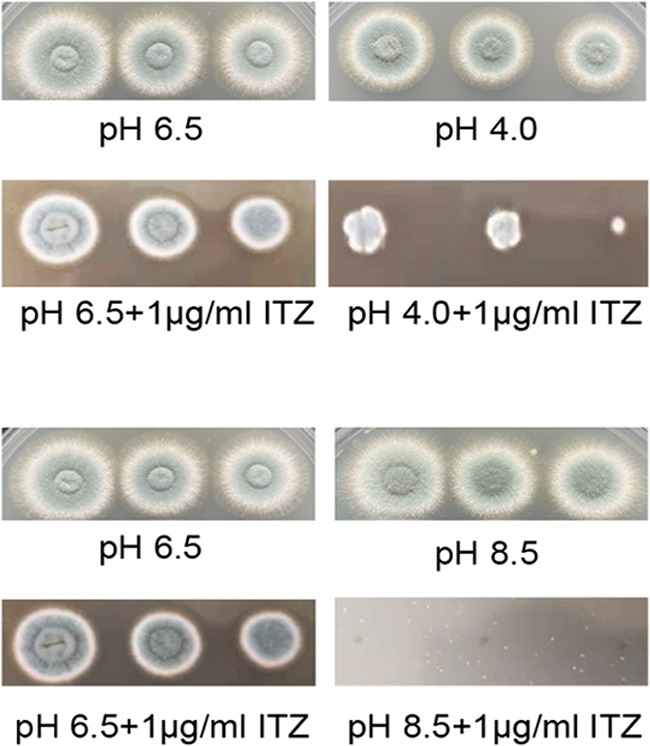
Acidic/alkaline pH stress affects the sensitivity of A. fumigatus to azole drugs. A. fumigatus is exquisitely sensitive to the combination of acidic/alkaline pH and azole drugs. A. fumigatus strains were inoculated as a series of 3-μL 10-fold dilutions derived from a starting suspension of 10^7^ conidia per mL onto solid YAG medium (pH 6.5, pH 4.0, or pH 8.5) with or without ITZ and were cultured at 37°C for 2 days.

Second, we tested the levels of killing resulting from a combination of acidic/alkaline pH (4.0 or 8.5) and azole drug (1 μg/mL) stresses in YAG medium. As shown in Fig. 2, A. fumigatus was more susceptible to itraconazole (ITZ) treatment at acidic pH than at normal pH. Interestingly, our results showed that A. fumigatus was hypersensitive to the combination of alkaline pH and ITZ stresses. Moreover, A. fumigatus was also hypersensitive to ITZ treatment at alkaline pH in MM (see Fig. S1C). Thus, simultaneous exposure to a combination of acidic/alkaline pH and azole drug stress killed wild-type A. fumigatus cells much more effectively than the corresponding individual stresses, indicating that these stresses might act synergistically. Previously, some results demonstrated that antifungal compounds trigger the intracellular accumulation of ROS in fungal pathogens and the induction of ROS production contributes to the ability of antifungal compounds to inhibit fungal growth (12). Thus, we measured the endogenous ROS levels in A. fumigatus in pH ranges of 4 to 8.5 with ITZ treatment. Our results showed that the ROS production in A. fumigatus treated with ITZ at acidic pH was significantly higher than that in A. fumigatus treated with ITZ at normal pH (see Fig. S2). Since intracellular ROS accumulation can lead to oxidative damage and lipid peroxidation in A. fumigatus, we speculated that lipid peroxidation induced by the elevated ROS accumulation might be the reason why A. fumigatus was sensitive to the combination of acidic pH stress and ITZ stress. However, the ROS production in A. fumigatus at alkaline pH was lower than that in A. fumigatus at normal pH (see Fig. S2). Additionally, under ITZ stress, the transcription levels of *catA*, *catB*, and *catC* significantly increased in A. fumigatus treated with alkaline pH, compared with A. fumigatus at normal pH (pH 6.5) (see Fig. S3). This result explains why ROS levels in A. fumigatus decreased during ITZ treatment at alkaline pH. Taken together, these results indicated that the hypersensitivity of A. fumigatus to the combination of alkaline pH stress and ITZ stress might not be related to ROS accumulation, and other molecular mechanisms might be involved in this process.

Next, we explored the molecular basis of the sensitivity of A. fumigatus to azole drugs at acidic/alkaline pH by genome-wide expression profiling. Wild-type A. fumigatus cells were exposed to acidic pH (pH 4.0) or alkaline pH (pH 8.5) for 1 h, harvested, and subjected to microarray analysis. Gene set enrichment analysis was performed to identify the pathways most affected by acidic/alkaline pH stress. Because of the large number of differentially expressed transcripts identified in this analysis, we performed a modified Kyoto Encyclopedia of Genes and Genomes (KEGG) analysis using FungiDB (https://fungidb.org/fungidb/app) and genes with a ≥2-fold change in transcript abundance in A. fumigatus treated with acidic/alkaline pH, compared with A. fumigatus at normal pH. Genes repressed in A. fumigatus treated at acidic pH are involved in biological processes including sphingolipid metabolism, glycerolipid metabolism, glycosphingolipid biosynthesis, fatty acid degradation, glycolysis/gluconeogenesis, and amino acid metabolism. Of the 26 genes that were downregulated in response to acidic pH stress, 7 were involved in lipid metabolism and 6 in amino acid metabolism (see Fig. S4A). These results indicate that acidic treatment of A. fumigatus caused the processes of lipid metabolism and amino acid metabolism to be greatly reduced. Furthermore, genes that were induced in acid-treated A. fumigatus were involved in glycosphingolipid biosynthesis, steroid biosynthesis, and fatty acid degradation. As shown in Table S1 in the supplemental material, microarray experiments revealed significant numbers of genes related to lipid metabolism and amino acid metabolism. Previous studies reported that amino acid metabolism and lipid metabolism, such as sphingolipid metabolism, are closely related to the regulation of azole sensitivity (5, 13–18). Therefore, we speculate that the increased sensitivity of A. fumigatus to azole drugs under acidic conditions may be related to a change of cell membrane lipid composition.

In exploring the mechanisms by which alkaline pH stress enhanced the susceptibility of A. fumigatus to azole drugs, our KEGG analysis showed that categories of downregulated genes in alkaline-treated A. fumigatus included sphingolipid metabolism, DNA repair, DNA replication, meiosis, cell cycle, ribosome, ribosome biogenesis, and amino acid metabolism (see Fig. S4B). These results indicate that alkaline treatment of A. fumigatus caused translation and processes related to genetic stability to be greatly reduced. As shown in Table S2, microarray experiments revealed 54 downregulated genes related to gene stability and lipid biosynthesis. Genetic stability is essential for the survival and maintenance of living organisms, and a very complex set of genes has evolved to repair damaged DNA. Recent studies showed that evolution of fungal azole drug resistance is associated with genetic stability (19–21). Additionally, previous studies reported that sphingolipid metabolism is closely related to the regulation of azole sensitivity (5, 22). Taken as a whole, the sensitivity of A. fumigatus to azole drugs under alkaline conditions may be related to a change of genetic stability and cell membrane lipid composition. Of course, it is necessary to further study how these related genes regulate azole resistance under alkaline conditions in the future.

In this study, we have addressed the impact of combined acidic/alkaline pH stress plus oxidative or azole drug stress on A. fumigatus. However, we predict that additional environmental cues, such as cationic stress or nutrient availability, might also influence combinatorial stress outputs in this fungus. This study provides a platform for future studies that will address the combined impacts of various environmental conditions and additional stresses on the adaptive responses of A. fumigatus and other pathogenic microbes.

### Data availability.

The RNA sequencing data have been deposited in the NCBI BioProject database under accession numbers PRJNA789093 and PRJNA789032.
