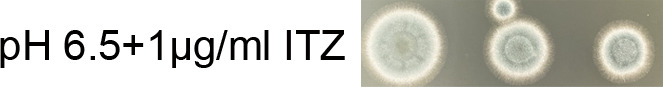# Correction for Song et al., “Acidic/Alkaline Stress Mediates Responses to Azole Drugs and Oxidative Stress in *Aspergillus fumigatus*”

**DOI:** 10.1128/spectrum.02996-23

**Published:** 2024-01-30

**Authors:** Jinxing Song, Landan Shi, Sha Wang, Yunqiu Wang, Yi Zhu, Jihong Jiang, Rongpeng Li

## AUTHOR CORRECTION

Volume 10, no. 1, e01999-21, 2022, https://doi.org/10.1128/spectrum.01999-21.

Page 3, Fig. 1A: In the YAG group, the image labeled “pH9.5” is a duplicate of the image labeled “pH8.5.” The correct image labeled “pH9.5” is shown in this Author Correction.


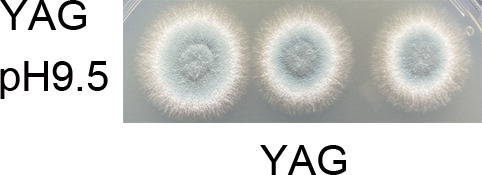


Page 3, Fig. 1A: The image labeled “pH4.0” in the YAG group is a duplicate of the image labeled “pH 4.0” in Fig. 2. The correct image labeled “pH4.0” in the YAG group is shown in this Author Correction.


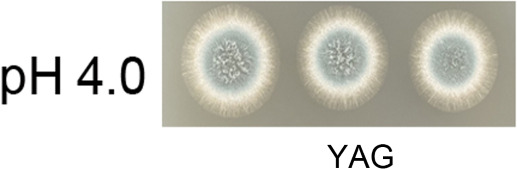


Page 4, Fig. 2: The image labeled “pH 6.5+1µg/ml ITZ” is duplicated. The correct image to replace one of the duplicated images is shown in this Author Correction.