# Supplementary material for: Acidic/Alkaline Stress Mediates Responses to Azole Drugs and Oxidative Stress in Aspergillus fumigatus
Source: Microbiol Spectr. 2022 Feb 23;10(1):e01999-21. doi: 10.1128/spectrum.01999-21 (PMC8865478; doi:10.1128/spectrum.01999-21)
Supplement: SUPPLEMENTAL FILE 1 — Supplemental material. Download SPECTRUM01999-21_Supp_1_seq6.pdf, PDF file, 0.7 MB [file spectrum01999-21_supp_1_seq6.pdf]

MATERIALS AND METHODS

Strains and culture conditions. *A. fumigatus* strain 1160 was purchased from the FGSC (Fungal Genetics Stock Center). Since the strain A1160 harbors a nonfunctional *pyrG* gene, the *Neurospora crassa pyr4* gene was complemented into the strain A1160, yielding the strain $A1160^C$ which harbors a functional *pyr4* gene. The media used in this study included YAG (2% glucose, 0.5% yeast extract, and trace elements), and MM (1% glucose, trace elements, and salts). 100 ml trace elements (2.20 g $ZnSO_4.7H_2O$, 1.10 g H_3BO_3, 0.50 g $MnCl_2.4H_2O$, 0.16 g $FeSO_4$. $7H_2O$, 0.16 g $CoCl_2.5H_2O$, 0.16 g $CuSO_4.5H_2O$, 0.11 g $(NH_4)_6Mo_7O_{24}.4H_2O$, 5.00 g Na_4EDTA). Media were adjusted to the required pH with HCl and NaOH as previously described by Taiki et al (1), and *A. fumigatus* strain was cultured on YAG or MM at 37 ℃.

Measurement of reactive oxygen species. Measurement of intracellular ROS production was performed by staining *A. fumigatus* hyphae with 2′,7′-dichlorodihydrofluorescein diacetate (H2DCFDA; Invitrogen) as previously described by Zhai et al with slight modifications (2). A suspension of 10^5 spores/ml diluted in YAG was dispensed into a 96-well plate, followed by incubation overnight at 37 ℃. Then, YAG was removed, and 2μg/ml concentration of itraconazole dissolved in YAG with specific pH was added per well, followed by incubation for 3 h. After a washing step with YAG, the cells were 10 μM 2′,7′-dichlorodihydrofluorescein diacetate at 37 ℃ for 45 min in the dark. To remove excess unreacted fluorescent probe, cells were washed with warm phosphate-buffered saline (PBS) at least three times. The fluorescence intensity was measured with an excitation filter at 485 nm and an emission filter at 530 nm, and unstained cells were used as a blank.

RNA extraction and RT-PCR. To analyze the relative expression levels of *catA*, *catB* and *catC* genes during itraconazole treatment at alkaline pH, *A. fumigatus* strain were incubated in YAG (pH6.5 or pH8.5) for 18 h at 37℃, and then the samples were supplemented with 2μg/ml itraconazole for 1 h. The samples were collected and subsequently frozen using liquid nitrogen. Total RNA was isolated using UNIQ-10 column total RNA purification kit (Shanghai Sangon Biotech) according to the manufacturer's instructions. For gDNA digestion and cDNA synthesis, the HiScriptII Q RT SuperMix for qRCR (+gDNA wiper) kit (Vazyme) were used according to the manufacturer's instructions, and then cDNA was used for the real-time analysis. Real-time PCRs were performed in triplicates, and the expression levels of all genes of interest were normalized to ß–tubulin levels, and expression levels were calculated using the ΔΔC T method.

RNA-seq. For RNA sequencing, *A. fumigatus* strain was grown in YAG and then exposed to acidic pH (pH 4.0) or alkaline pH (pH 8.5) for 1h. The samples were collected and subsequently frozen using liquid nitrogen. After mRNA purification and library construction, the samples were sequenced by next-generation sequencing (NGS) based on the Illumina NextSeq 500 sequencer sequencing platform. RNA isolation, mRNA purification, and cDNA synthesis and sequencing were performed by Wuhan Benagen Tech Solutions Company Limited (China).

REFERENCES

1. Futagami T, Nakao S, Kido Y, Oka T, Kajiwara Y, Takashita H, Omori T, Furukawa K, Goto M. 2011. Putative stress sensors WscA and WscB are involved in hypo-osmotic and acidic pH stress tolerance in Aspergillus nidulans. Eukaryot Cell 10:1504-15.
2. Zhai PF, Shi LD, Zhong GW, Jiang JH, Zhou JW, Chen X, Dong GK, Zhang L, Li RP, Song JX. 2021. The OxrA Protein of Aspergillus fumigatus Is Required for the Oxidative Stress Response and Fungal Pathogenesis. Applied and Environmental Microbiology 87.

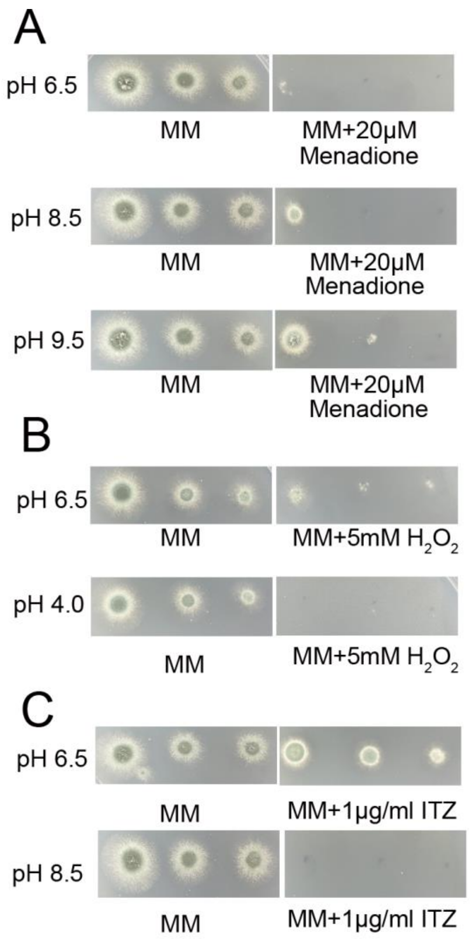

Fig. S1 Acidic/alkaline pH stress affects the sensitivity of *A. fumigatus* to oxidative stress and azole drugs in MM medium. (A) *A. fumigatus* is resistant to the combination of alkaline pH and menadione. *A. fumigatus* strains were inoculated as a series of 3-μl 10-fold dilutions derived from a starting suspension of 10^7 conidia per ml on to solid MM (pH 6.5, pH 8.5 or pH 9.5) with or without 20 μM menadione and cultured at 37 ℃ for 2 days. (B) *A. fumigatus* is exquisitely sensitive to the combination of acidic pH and oxidative stresses. *A. fumigatus* strains were inoculated as a series of 3-μl 10-fold dilutions derived from a starting suspension of 10^7 conidia per ml on to solid MM (pH 6.5 or pH 4.0) with or without 5 mM H_2O_2 and cultured at 37 ℃ for 2 days. (C) *A. fumigatus* is exquisitely sensitive to the combination of alkaline pH and azole drugs.

A. fumigatus strains were inoculated as a series of 3-μl 10-fold dilutions derived from a starting suspension of 10^7 conidia per ml on to solid MM (pH 6.5, pH 4.0) with or without ITZ and cultured at 37 ℃ for 2 days.

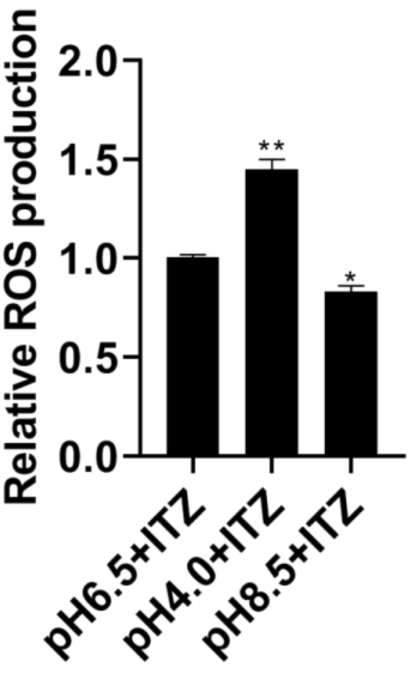

Fig. S2 The endogenous ROS levels in *A. fumigatus* in pH ranges of 4-8.5 with itraconazole treatment. The ROS contents of *A. fumigatus* treated with itraconazole at acidic/alkaline pH were normalized to that of *A. fumigatus* treated with itraconazole at normal pH. The experiment was performed thrice with biological triplicates. The data are presented as the means and standard deviations of three biological replicates. Statistical analysis was performed using an unpaired two-tailed t test (**, $P < 0.01$).

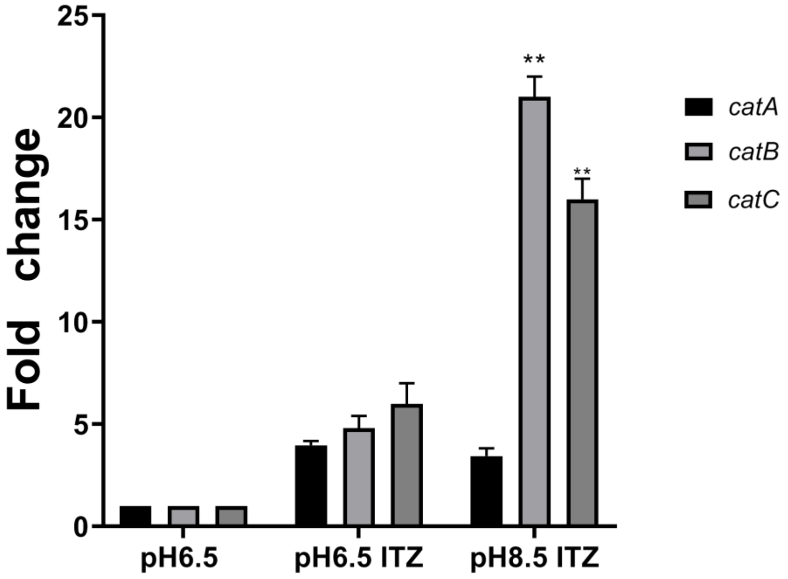

Fig. S3 The catalase expression levels during itraconazole treatment at alkaline pH in *A. fumigatus*. Quantitative real-time RT-PCR of *catA*, *catB* and *catC* transcript levels during itraconazole treatment at alkaline pH in *A. fumigatus*. *A. fumigatus* strain were incubated in YAG (pH6.5 or pH8.5) for 18 h at 37℃, and then the samples were supplemented with 2μg/ml itraconazole for 1 h. Gene expression was normalized to the endogenous reference gene *tubA*. Experiments were carried out in triplicate. Values are reported as the means ± standard errors of the means (SEM). Statistical significance was calculated using the unpaired two-tailed t test (ns, not significant).

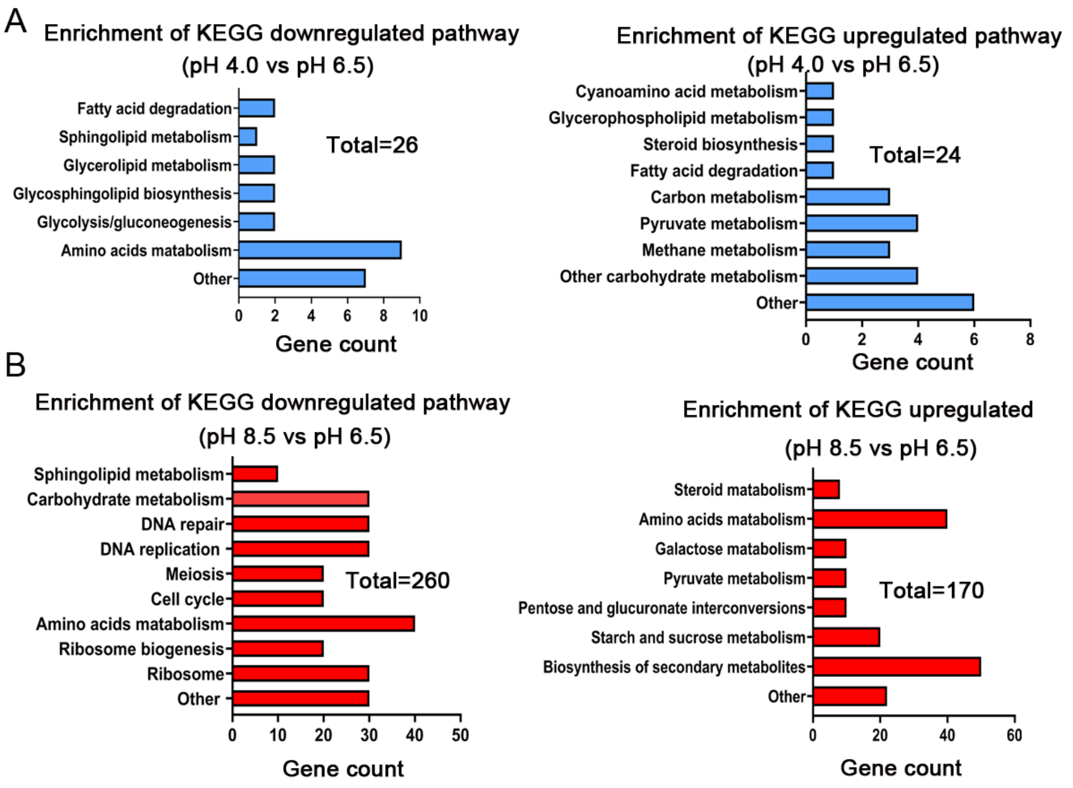

Fig. S4 Acidic or alkaline pH results in significant changes in the patterns of gene transcription. (A) Wild-type *A. fumigatus* cells were exposed to acidic pH (pH 4.0) or alkaline pH (pH 8.5) for 1h. Total RNA was extracted, mRNA was isolated, and libraries were prepared and finally sequenced using an Illumina NextSeq 500 sequencer. KEGG analysis of *A. fumigatus* strains at pH 4.0 versus *A. fumigatus* strains at pH 6.5 differentially expressed genes. These transcripts were selected based on a strict cutoff of log 2 fold change of ±1. (B) KEGG analysis of *A. fumigatus* strains at pH 8.5 versus *A. fumigatus* strains at pH 6.5 differentially expressed genes. These transcripts were selected based on a strict cutoff of log 2 fold change of ±1.

Table S1 Selected significant genes which may contribute to drug resistance in acidic-treated *A. fumigatus*

Lipid Metabolism	Description	Fold changes (log 2)
AFUA_7G00200	Predicted transferase activity, lipid metabolic	-2.2
AFUA_5G13830	hydrolase activity, sphingolipid metabolism	-2.4
AFUA_8G05020	N-acetylhexosaminidase, glycosphingolipid biosynthesis	-2.8
AFUA_8G01130	Alpha-galactosidase, glycosphingolipid biosynthesis	-4.1
AFUA_3G00290	Lipid metabolic process,predicted oxidoreductase activity	-3.6
AFUA_7G01010	Putative alcohol dehydrogenase, fatty acid degradation	-3.5
AFUA_8G02380	Dehydroaustinol biosynthetic process, lipid metabolic	-2.5
AFUA_3G14680	Putative secreted lysophospholipase B, Glycerophospholipid metabolism	3.8
AFUA_4G09190	Putative S-adenosyl-methionine-sterol-C methyltransfera -se	4.0
AFUA_2G01040	Putative formaldehyde dehydrogenase	3.6
**Amino acid metabolism**		
AFUA_7G02420	Malate dehydrogenase, amino acid metabolic process	-2.3
AFUA_7G01000	Putative alcohol dehydrogenase, amino acid metabolism	-3.5
AFUA_8G07330	Predicted NAD binding, amino acid metabolism	-3.4
AFUA_4G03710	Predicted catalytic activity, alanine, aspartate and glutamate metabolism	-3.1
AFUA_7G01010	Putative alcohol dehydrogenase, tyrosine metabolism	-3.5
AFUA_4G03700	Nuclear periphery localization, purine metabolism	-3.2

Table S2 Selected downregulated genes which may contribute to drug resistance in alkaline-treated *A. fumigatus*

DNA replication	Description	Fold changes (log 2)
AFUA_2G12250	DNA strand elongation involved in DNA replication	-2.6
AFUA_3G09020	Nuclear chromatin, nucleus localization, DNA replication	-2.4
AFUA_2G05570	Predicted role in RNA catabolic process	-2.6
AFUA_3G06060	5'-3' exodeoxyribonuclease activity	-2.5
AFUA_3G14010	DNA replication origin binding	-2.2
AFUA_5G02520	Single-stranded DNA helicase activity	-2.2
AFUA_4G07970	DNA-directed DNA polymerase activity	-2.1
AFUA_6G10560	DNA-directed DNA polymerase activity	-2.0
AFUA_2G10140	3'-5' DNA/RNA helicase activity	-1.9
**Base excision repair**		
AFUA_3G06060	5'-3' exodeoxyribonuclease activity	-2.5
AFUA_4G07970	DNA-directed DNA polymerase activity	-2.1
AFUA_6G10560	DNA-directed DNA polymerase activity	-2.0
AFUA_5G07320	Putative polyADP-ribose polymerase (PARP)	-2.0

**Mismatch repair**		
AFUA_2G12250	DNA strand elongation involved in UV-damage excision repair	-2.6
AFUA_4G07970	DNA-directed DNA polymerase activity	-2.1
AFUA_2G03910	Ortholog(s) have role in cellular response to UV	-2.1
AFUA_6G10560	DNA synthesis involved in UV-damage excision repair	-2.0
**Cell Cycle**		
AFUA_1G14730	Anaphase-promoting complex binding	-3.3
AFUA_3G06200	Anaphase-promoting complex localization	-3.5
AFUA_6G09180	Ortholog(s) have protein kinase activator activity	-2.7
AFUA_6G08200	Ortholog(s) have phosphoprotein phosphatase activity	-2.5
AFUA_3G14010	Ortholog(s) have DNA replication origin binding	-2.2
AFUA_1G02500	DNA replication origin binding activity, role in mitotic DNA replication initiation	-2.2
AFUA_5G02520	Ortholog(s) have DNA replication origin binding, chromatin binding	-2.2
AFUA_2G01520	Ortholog(s) have role in ascospore formation	-2.4
AFUA_3G08280	Ortholog(s) have anaphase-promoting complex binding, cyclin binding	-2.1
AFUA_1G15710	Ortholog(s) have role in anaphase-promoting complex-dependent catabolic process	-2.2
AFUA_4G06020	Putative cyclin dependent kinase inhibitor	-2.1
AFUA_2G10140	Ortholog(s) have 3'-5' DNA/RNA helicase activity	-2.0
AFUA_4G12670	Ortholog(s) have role in intra-S DNA damage checkpoint	-2.0
**Meiosis**		
AFUA_1G14730	Ubiquitin ligase activator activity	-3.3
AFUA_3G06200	Ortholog(s) have anaphase-promoting complex localization	-3.5
AFUA_6G09180	Ortholog(s) have protein kinase activator activity	-2.7
AFUA_2G13140	A serine/threonine protein kinase	-2.4
AFUA_3G14010	Ortholog(s) have DNA replication origin binding	-2.2
AFUA_1G02500	Ortholog(s) have DNA replication origin binding activity	-2.2
AFUA_5G02520	Ortholog(s) have DNA replication origin binding	-2.2
AFUA_1G15710	Ortholog(s) have role in anaphase-promoting complex-dependent catabolic process	-2.2
AFUA_2G10140	Ortholog(s) have 3'-5' DNA/RNA helicase activity	-2.0
AFUA_4G12670	Ortholog(s) have role in intra-S DNA damage checkpoint	-2.0
**Nucleotide excision repair**		
AFUA_2G12250	Ortholog(s) have role in DNA strand elongation	-2.6
AFUA_4G07970	Ortholog(s) have DNA-directed DNA polymerase activity	-2.1
AFUA_2G03910	Ortholog(s) have role in cellular response to UV	-2.1
AFUA_6G10560	Ortholog(s) have DNA-directed DNA polymerase activity	-2.0
AFUA_5G01800	DNA repair protein	-1.9
AFUA_4G08900	Ortholog(s) have cyclin-dependent protein	-1.9

	serine/threonine kinase activator activity	
**Sphingolipid metabolism**		
AFUA_1G11890	Putative serine palmitoyltransferase 2 lcb2	-2.5
AFUA_1G16850	Putative sphinganine hydroxylase sur2	-2.5
AFUA_6G10460	Putative ceramide synthase membrane component lag1	-2.2
AFUA_4G06290	Ortholog(s) have sphingosine N-acyltransferase activity	-2.1
AFUA_4G06940	Ortholog(s) have sphingolipid delta-4 desaturase activity	-2.1
**Steroid biosynthesis**		
AFUA_1G07140	Putative C-24(28) sterol reductase erg4	-3.0
AFUA_1G04720	C-8 sterol isomerase erg2	-2.2
AFUA_1G03950	Sterol C22 desaturase erg5	-2.0
